# Involvement of L polymerase and heat shock proteins in the biogenesis of viral circular RNAs derived from respiratory syncytial virus

**DOI:** 10.1128/mbio.03980-25

**Published:** 2026-02-12

**Authors:** Mingzhen Lin, Shanshan Miao, Xinru Yang, Yezhenghong Qiu, Zesen Mai, Yao Xiao, Mengyuan Xie, Yulong Luo, Caiqi Ma, Jifang Liu, Zhaoyu Liu, Wenxia Yao

**Affiliations:** 1 Key Laboratory of Biological Targeting Diagnosis, Therapy and Rehabilitation of Guangdong Higher Education Institutes, GuangDong Engineering Technology Research Center of Biological Targeting Diagnosis, Therapy and Rehabilitation, State Key Laboratory of Respiratory Disease, The Fifth Affiliated Hospital, Guangzhou Medical University, The Fifth Clinical College of Guangzhou Medical University 26468, Guangzhou, China; 2 Shenzhen Nanshan People’s Hospital, Shenzhen, Guangdong, China; Johns Hopkins University Bloomberg School of Public Health, Baltimore, Maryland, USA

**Keywords:** RSV, viral circRNAs, biogenesis, L polymerase, HSP70, HSP90, inclusion bodies

## Abstract

Circular RNAs (circRNAs) are new members of noncoding RNAs that have gained increased attention, and reports on viral circRNAs and their functions continue to emerge in recent years. However, the mechanisms underlying the biogenesis of viral circRNAs, especially those produced by RNA viruses, remain largely unexplored. Our recent research systematically identified and characterized viral circRNAs induced by respiratory syncytial virus (RSV), a human RNA virus for which no effective treatments exist. In this study, we further investigated the mechanism underlying the production of RSV circRNAs. We found that the viral L polymerase and the cellular HSP70 and HSP90 proteins associate with viral circRNAs through proteomic analysis of circRNA pulled-down lysates. These interactions were further confirmed using RNA-binding protein immunoprecipitation assays. We also observed that RSV circRNAs colocalize with the L polymerase and HSP70/90 proteins within the synthesis site, inclusion bodies (IBs). Moreover, both the viral L protein and the cellular HSP70 and HSP90 proteins modulate the production of viral circRNAs. Finally, the flanking AT/TA sequences of RSV circRNAs serve as *cis*-acting elements. Collectively, our study demonstrates that L polymerase and HSP70/90 proteins, identified as novel RSV circRNA-associated proteins, contribute to the biogenesis of viral circRNAs. This study provides new insights into host-RSV interactions and establishes a foundation for the development of innovative anti-RSV strategies.

**IMPORTANCE:**

As an RNA virus, respiratory syncytial virus (RSV) imposes a serious global disease burden, and effective treatments for RSV are still lacking. An increasing number of research studies has discovered and validated the presence of viral circular RNAs (circRNAs) and their functions in viral infection. However, elucidating the biogenesis of viral circRNAs, particularly those of RNA viruses, remains an intriguing challenge. In this study, we first explored the production mechanism of RSV circRNAs. We demonstrated that L polymerase and HSP70/90 proteins, identified as novel RSV circRNA-associated proteins, contribute to the biogenesis of viral circRNAs in inclusion bodies (IBs). Our study provides a comprehensive understanding of the crosstalk among RSV circRNA, L polymerase, HSP70/90 proteins, and IBs, which is essential for identifying novel anti-RSV strategies.

## INTRODUCTION

Coronaviruses and other RNA viruses cause a variety of infectious diseases, many of which can lead to global pandemics. As an RNA virus, respiratory syncytial virus (RSV) imposes a serious disease burden globally. RSV is the leading cause of severe respiratory infections in infants and young children (1). It also poses a significant burden on the elderly, asthmatic and immunocompromised patients, and other at-risk populations (2). Despite the availability of three vaccines and two prophylactic monoclonal antibodies, there remains an unmet medical need for RSV treatment (3). Enhancing research on RSV-host interactions can significantly aid in developing prevention and control strategies for RSV.

RSV is a non-segmented negative-sense RNA virus (nsNSV, also termed Mononegavirales) that belongs to the genus *Orthopneumovirus* within the family *Pneumoviridae* (4). Its 15.2 kb genomic RNA contains 10 genes in the order NS1-NS2-N-P-M-SH-G-F-M2-L encoding a total of 11 proteins. Similar to other nsNSV, the RSV genome is tightly bound to the viral nucleoprotein N and serves as a template for two processes: transcription that generates mRNAs and replication that yields an antigenome or genome. The replication of antigenome/genome and mRNA transcription are collectively referred to as RNA synthesis. Almost all RNA viruses encode RNA-dependent RNA polymerase (RdRp). RSV also encodes RdRp, which mediates RSV RNA synthesis and is composed of the large polymerase L, the catalytic core, and its main cofactor, the phosphoprotein P (5). Aside from L and P, the RSV N and M2-1 proteins, along with the RNA genome, are virus-specific components required for RNA synthesis. Most RNA viruses encode only a small number of proteins. However, they can utilize their limited number of viral proteins to perform multiple functions through interaction and coordination with host cellular proteins (6). Cellular proteins have been shown to play crucial roles in viral infection for many RNA viruses. These roles include participating in the formation of virus replication organelles or factories, as well as acting directly as chaperones or cofactors to regulate the activity of viral proteins. Heat shock proteins (HSPs) are highly conserved cellular chaperone proteins, which can be utilized by viruses to facilitate infection and replication (7). Based on the molecular weight, HSPs are classified into different families, including HSP90s and HSP70s. Infections by negative-strand RNA viruses (NSVs) lead to the formation of viral inclusion bodies (IBs) in host cells, which concentrate proteins to enable efficient viral replication (8). RSV IBs are described as spherical cytoplasmic structures to which all viral proteins of polymerase complex (N, P, L, and M2-1) are recruited for efficient viral transcription and replication (9). In addition to viral proteins, RSV IBs also recruit cellular proteins such as HSP90 and HSP70, which are involved in stabilizing the L protein and facilitating viral RNA synthesis (10, 11).

As a new member of the noncoding RNA (ncRNA) family, circular RNA (circRNA) is a closed circular RNA formed by the covalent linkage of 3′ and 5′ ends, resulting in no free ends (12, 13). The widespread use of high-throughput sequencing and rapid advancements in bioinformatics have led to the discovery of numerous circRNAs, which are promising biomarkers and therapeutic targets. CircRNAs are characterized by specific expression patterns and resistance to exonuclease activity, and they play important roles in various biological processes and disease development (14). In the field of viral biology, an increasing number of research teams have discovered and validated the presence of viral circRNAs across various viruses (15, 16), including early DNA viruses (17–21) and later RNA viruses (22–24). Multiple biological roles have been attributed to viral circRNAs, including the regulation of viral replication and persistence, as well as the encoding of oncoproteins (22, 25–27). Although evidence regarding viral circRNAs and their functions continues to emerge, the biogenesis mechanisms of most viral circRNAs remain largely unknown; in particular, clarifying the biogenesis of viral circRNA in RNA viruses poses an intriguing challenge. Interestingly, our research, along with the studies by Cao and Yang, suggests that circRNAs derived from certain RNA viruses, including RSV, hepatitis C virus (HCV), and betacoronaviruses, appear to be produced by mechanisms independent of U2 splicing (22–24), although the production of cellular circRNA is primarily associated with canonical back-splicing mechanisms (28).

Our recent research systematically identified and characterized viral circRNAs induced by RSV (23). In this study, we further explored the production mechanism of RSV circRNAs and investigated the proteins and sequence components required for circRNA production. In the current study, we found that the viral L polymerase and cellular HSP70 and HSP90 proteins associate with viral circRNAs. We also observed that RSV circRNAs colocalize with the L protein and HSP70**/**HSP90 proteins within synthesis site IBs. Furthermore, both the viral L polymerase and cellular HSP70 and HSP90 proteins regulate the production of viral circRNAs. Finally, the flanking AT/TA sequences of RSV circRNAs function as *cis*-acting elements. Overall, our study demonstrates that L polymerase and HSP70/90 proteins, identified as novel RSV circRNA-associated proteins, contribute to the biogenesis of viral circRNAs, revealing novel layers of host-RSV interactions.

## RESULTS

### Identification of L protein and HSPs as rsv_circ_RNA binding proteins by proteomics

**Fig 1:**
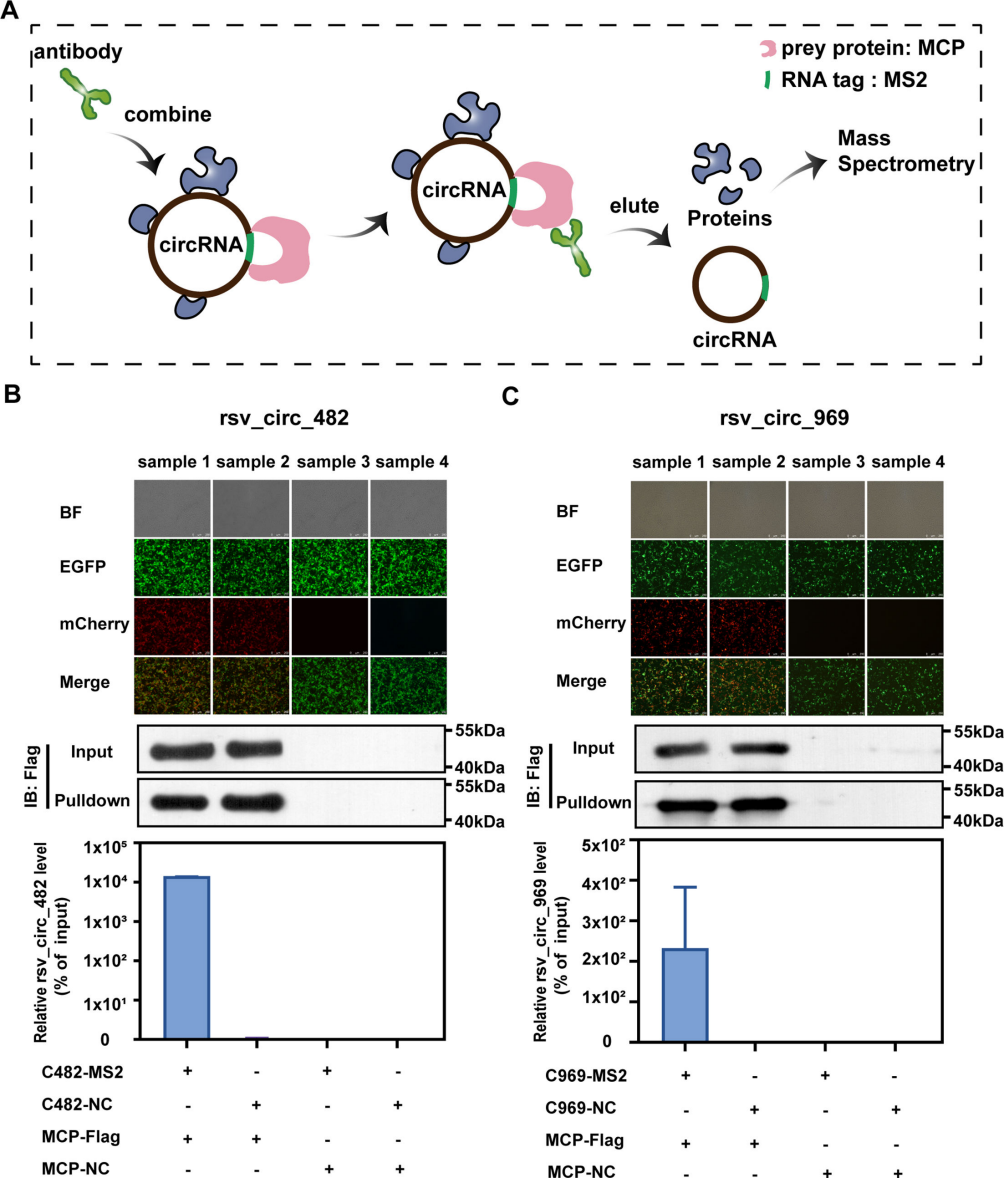
Identification of rsv_circ_RNA binding proteins by circRNA pull-down coupled proteomics. (**A–C**) Viral circRNA was engineered to contain RNA tag MS2 sequences, enabling the capture of circRNA-bound proteins in cellular lysates via the high-affinity interaction between the MS2 tag and MCP. MCP, MS2 capture protein. (**A**) Schematic of circRNA pull-down and the subsequent workflow for mass spectrometry (MS)-based proteomic identification of rsv_circ_RNA binding proteins. (**B and C**) HEK293T cells were co-transfected with RSV circRNA and MCP overexpression plasmids followed by RSV infection. The successful transfection of RSV circRNA and MCP protein was ascertained and visualized by co-expressed EGFP and mCherry autofluorescence, respectively (top panel). Pull-down assays with WB (middle panel) and qRT-PCR (bottom panel) showed that the prey protein and rsv_circ_482 (**B**) or rsv_circ_969 (**C**) were pulled down by MCP-Flag antibodies. BF: bright field; NC, negative control; C482-MS2, overexpression of rsv_circ_482 containing MS2 tag; C482-NC, overexpression of rsv_circ_482 without tag; C969-MS2, overexpression of rsv_circ_969 containing MS2 tag; C969-NC, overexpression of rsv_circ_969 without tag.

In order to gain a comprehensive understanding of the production mechanism of viral circRNA, pull-down assays coupled with mass spectroscopy (MS) proteomics were used to identify proteins that associate with circRNAs. This system utilized the overexpression of rsv_circ_482 or rsv_circ_969 in the context of RSV infection. Herein, we chose rsv_circ_482 and rsv_circ_969 as representatives of viral circRNAs due to their high expression in RSV-infected cells (23). Overexpression of rsv_circ_482 and rsv_circ_969 was engineered to contain RNA tag MS2 sequences, enabling the capture of rsv_circ_482/969-bound proteins in cellular lysates via the high-affinity interaction between the MS2 RNA tag and MS2 capture proteins (MCPs) bound to beads (Fig. 1A through C, sample 1). As controls for the capture, we used protein extracts from RSV-infected cell lines with overexpression of rsv_circ_482/969 without MS2 sequences (Fig. 1B and C, sample 2). Pull-down MS experiments were performed in duplicate, and the pulled-down proteins were subsequently analyzed using label-free MS.

As shown in Fig. 1B and C (top panels), we successfully transfected plasmids expressing viral circRNAs (co-expressed with EGFP label) and the MCP prey protein (co-expressed with mCherry label) into HEK293T cells. Moreover, western blotting (WB) results showed that MCPs were successfully pulled down with Flag-fused antibodies in both rsv_circ_482 and rsv_circ_969 experiments (Fig. 1B and C, middle panel). qPCR results showed that both rsv_circ_482 and rsv_circ_969 were pulled down with the Flag antibody as well (Fig. 1B and C, bottom panels).

Further MS results revealed the presence of the viral L polymerase in the protein complex pulled down by the viral circRNA. Five HSP70 isoforms (HSPA8, HSPA1A, HSPA1L, HSPA6, and HSPA2) and three HSP90 isoforms (HSP75, HSP90AB1, and HSPAA1) were also detected in the viral circRNA-bound protein complex (Table 1). These results suggest that the L polymerase and cellular HSP70/90 proteins associate with viral circRNAs to play a certain function. Among the HSP70 and HSP90 isoforms, HSP70 (HSPA8) and HSP90 (HSP90AB1) exhibited the highest protein scores in their respective families (Table 1) and were thus selected for further investigation.

**TABLE 1:** Partial proteins identified in the MS that interacted with rsv_circ_482/969^*a*^

Protein	Viral circRNA	Family	Prot_acc	Prot_desc	Prot_ score	Prot_ cover
Viral	rsv_circ_969	L protein	O09721|O09721_RSV	RNA-directed RNA polymerase L	14	0.3
	rsv_circ_482	HSP70	P11142|HSPA8_Human	Heat shock cognate 71 kDa protein	281	9.8
	rsv_circ_482	HSP70	P0DMV8|HSPA1A_Human	Heat shock 70 kDa protein 1 A	188	14.4
	rsv_circ_482	HSP70	P34931|HSPA1L_Human	Heat shock 70 kDa protein 1-like	152	8.1
	rsv_circ_482	HSP70	P17066|HSPA6_Human	Heat shock 70 kDa protein 6	120	6.2
Host	rsv_circ_482	HSP90	P08238|HSP90AB1_Human	Heat shock protein 90-beta	58	5.8
	rsv_circ_482	HSP90	P07900|HSP90AA1_Human	Heat shock protein HSP 90-beta	58	4.9
	rsv_circ_482	HSP90	Q12931|HSP75_Human	Heat shock protein 75 kDa	56	2
	rsv_circ_969	HSP70	P54652|HSPA2_Human	Heat shock-related 70 kDa protein 2	18	3.8
	rsv_circ_969	HSP70	P34931|HSPA1L_Human	Heat shock 70 kDa protein 1-like	18	3.7

^*a*^ Prot**_**acc, protein accession id；Prot**_**desc, protein description; Prot**_**score, protein score; Prot**_**cover, protein cover percentage.

### Validation of rsv_circ_482/969-protein interaction by RNA-binding protein immunoprecipitation (RIP)

**Fig 2:**
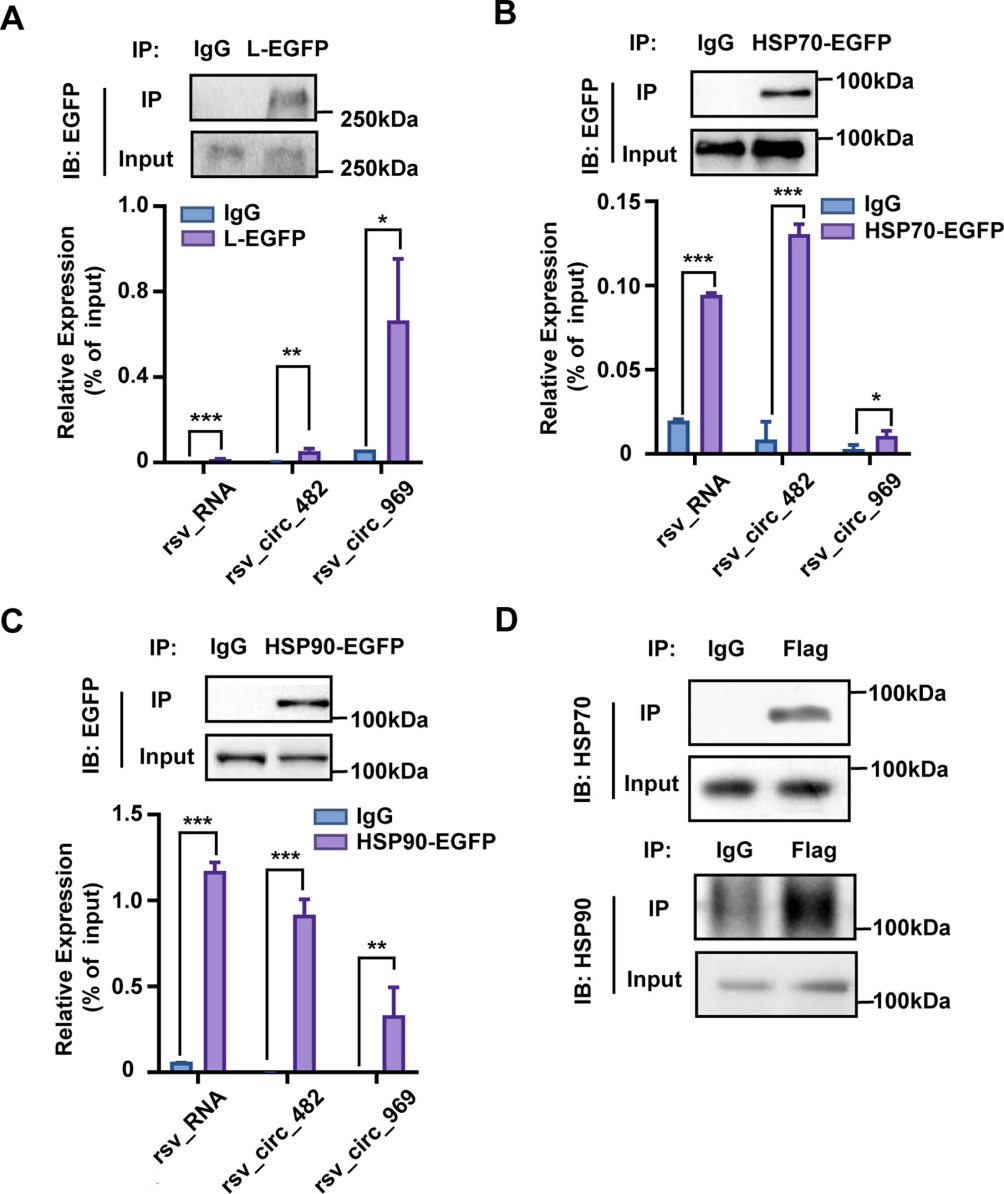
Validation of rsv_circ_482/969-protein interactions. (**A–C**) RIP assay was conducted to determine the rsv_circ_482/969-protein interaction. BHK21 cells were transfected with an L-EGFP (**A**) or HSP70-EGFP (**B**) or HSP90-EGFP (**C**) overexpressing plasmid, followed by inoculation with RSV viruses. Cell lysates were subjected to IP with anti-EGFP antibodies, and IgG was used as a negative control. The protein expressions of L/HSP70/HSP90-EGFP were analyzed by WB. The expression of rsv_RNA and rsv_circ_482/969 in different groups was detected by qRT-PCR. *, *P* < 0.05; **, *P* < 0.01; ***, *P* < 0.001. (**D**) HEK293T cells were transfected with rsv_circ_482 and MCP overexpression plasmids described in Fig. 1 and then infected with RSV. Pull-down assays coupled with WB were used to validate the interaction between rsv_circ_482 and HSP70/90 proteins.

To further validate our MS results, we examined the interactions of rsv_circ_482/969 with proteins using RIP. First, to investigate the interaction between rsv_circ_482/969 and the L polymerase, BHK21 cells were transfected with an L-EGFP-overexpressing plasmid, followed by inoculation with RSV viruses. RIP was then performed using anti-EGFP antibodies. RSV RNA, which relies on interaction with L protein for its synthesis, was used as a control. As expected, L-EGFP protein was immunoprecipitated by anti-EGFP antibodies, and RSV RNA was enriched in the L-EGFP immunoprecipitates (Fig. 2A). Under these conditions, rsv_circ_482 was significantly enriched in the RNA co-precipitated with anti-L-EGFP antibody (Fig. 2A), and rsv_circ_969 showed similar enrichment (Fig. 2A). Almost no RNAs were immunoprecipitated by the negative IgG controls. These results validate the interaction between rsv_circ_482/969 and L polymerase, as demonstrated by the RIP assay.

We further confirmed the interaction between rsv_circ_482/969 and HSP70/HSP90s through RIP experiments. In a similar approach, BHK21 cells were transfected with HSP70-EGFP or HSP90-EGFP overexpression plasmids and subsequently infected with RSV. WB analysis following RIP revealed the immunoprecipitation of HSP70/90 proteins, and qPCR analysis after RIP confirmed the interaction between rsv_circ_482/969 and HSP70/HSP90 (Fig. 2B and C). In addition, pull-down assays coupled with WB were used to confirm the interaction between rsv_circ_482 and HSP70/90 (Fig. 2D).

Collectively, these results further confirm that rsv_circ_482/969 associates with L polymerase and HSP70/HSP90 proteins.

### RSV viral circRNAs colocalize with L and HSP70/HSP90 within synthesis site IBs

It is now well-known that RSV RNA is found to be produced in “RSV factories” IBs (8). The study by Rincheval et al. (9) demonstrated that IBs are sites of viral RNA synthesis by analyzing 5-ethynyl-uridine (5EU) incorporation into nascent viral RNAs. Since 5EU does not differentiate between mRNA, circRNA, and genomic RNA, we extend the concept of viral RNA synthesis to include circRNA synthesis and thus infer that IBs may also serve as synthesis sites of RSV circRNAs.

**Fig 3:**
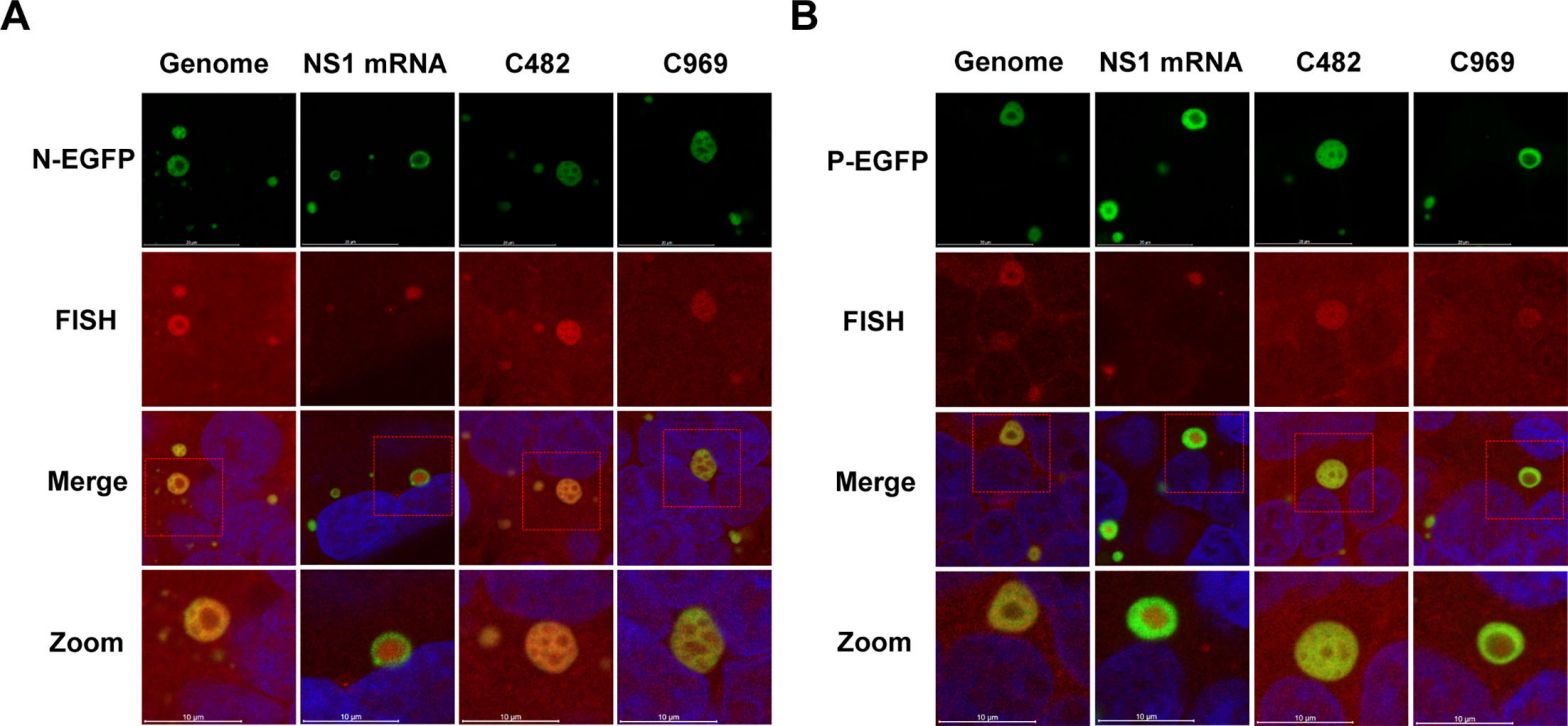
Localization of viral circRNAs in RSV-induced IBs. HEK293T cells were transfected with plasmids encoding the N-EGFP (**A**) or P-EGFP (**B**) proteins and then infected with RSV for 48 h. The expression of N-EGFP and P-EGFP proteins was visualized by their spontaneous green fluorescence, which indicates spherical IBs in RSV-infected cells. FISH analyses were performed with specific probes (red) to detect viral genomic RNA, NS1 mRNA, rsv_circ_482 (abbreviated as C482), or rsv_circ_969 (abbreviated as C969) as indicated on the pictures. Cells were also stained with DAPI (merge). Images were taken under a confocal microscope and representative images are shown (taken in triplicate). The boxed areas enclose IBs that are shown magnified (zoom). Scale bar: 10 µm.

In order to determine whether IBs are sites of viral circRNA synthesis, we performed circRNA staining in RSV-infected cells. The N and P proteins are reported as the basic scaffold for IB formation during RSV infection (29); therefore, we used N or P proteins to indicate IB in RSV-infected cells. Fluorescent proteins (EGFP) were fused and used to visualize N or P proteins. Cells were transfected with N-EGFP or P-EGFP plasmids and then infected with RSV. Forty-eight hours post-infection (hpi), cells were examined with N-EGFP or P-EGFP autofluorescence to detect IBs. They were then prepared for FISH using Cy3-labeled oligonucleotide probes designed to specifically detect either viral rsv_circ_482 or rsv_circ_969. The previously reported probes targeting genomic RNA and NS1 mRNA (9) were used as controls. Results showed that, like genomic RNA and NS1 mRNA, FISH signals for rsv_circ_482 and rsv_circ_969 localized within the N-EGFP- or P-EGFP-indicated IBs (Fig. 3A and B), confirming that IBs are sites of RSV circRNA synthesis.

**Fig 4:**
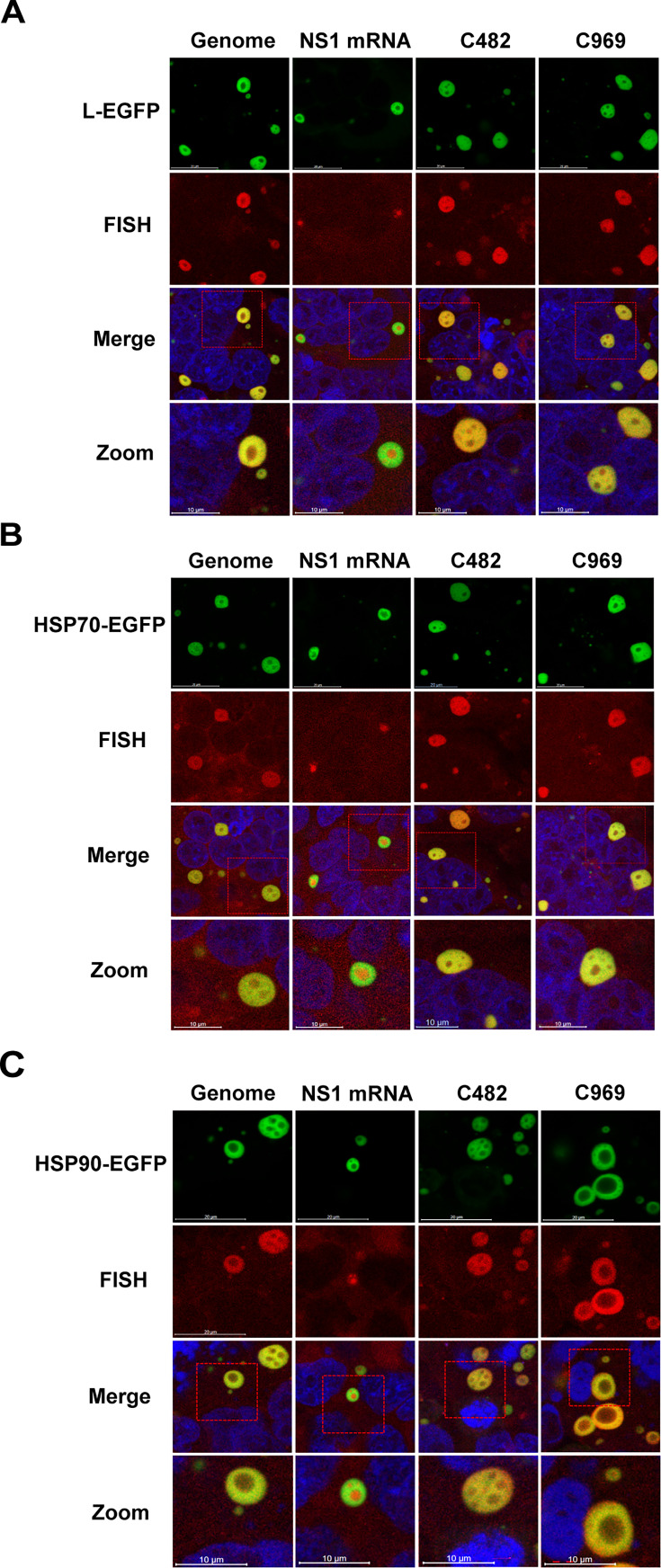
Co-localization of L polymerase and HSP70/HSP90 proteins with viral circRNAs inside RSV-induced IBs. HEK293T cells were transfected with plasmids encoding the L-EGFP (**A**) or HSP70-EGFP (**B**) or HSP90-EGFP (**C**) and then inoculated with RSV. The expressed L-EGFP/HSP70-EGFP/HSP90-EGFP proteins are visualized by their autofluorescence in the first line. FISH analyses were performed to detect viral genomic RNA, NS1 mRNA, rsv_circ_482 (abbreviated as C482), or rsv_circ_969 (abbreviated as C969) (in red) and were also stained with DAPI (merge). Co-localization was determined by the yellow signal from the merged images. Representative images were taken under a Zeiss LSM 800 confocal microscope (taken in triplicate). The framed sections enclose IBs, which are shown magnified (zoom) in the fourth line. Scale bar: 10 µm.

The aforementioned results by MS (Fig. 1) and RIP (Fig. 2) indicated that the viral L protein and cellular HSP70/90 proteins interact with viral circRNAs. Interestingly, both the L and HSP70/90 proteins are documented to be recruited to IBs, where they participate in viral RNA synthesis (8–10). Herein, we further determined whether RSV circRNAs co-localized with RSV-L and HSP70/90 proteins in the synthesis site IBs, thereby participating in circRNA synthesis. Cells were transfected with either L-EGFP plasmids or HSP70/90-EGFP plasmids, followed by infection with RSV. Subsequently, FISH experiments were performed. As shown in Fig. 4, viral genomic RNA and NS1 mRNA were detected to colocalize with L and HSP70/HSP90 in IBs. Similarly, viral circRNAs were also found to accumulate in IBs with L and HSP70/HSP90 (Fig. 4). These results suggest that RSV circRNAs colocalize with L and HSP70/HSP90 in IBs and are involved in circRNA synthesis.

Taken together, these results suggest that the biogenesis of RSV circRNA occurs in IBs, which contain or recruit proteins necessary for viral circRNA synthesis. Since L polymerase and HSP70/90 proteins associate with RSV circRNAs within active synthesis compartments IBs, we further investigate whether interfering with their expression affects viral circRNA generation.

### Viral L protein is involved in viral circRNA production

**Fig 5:**
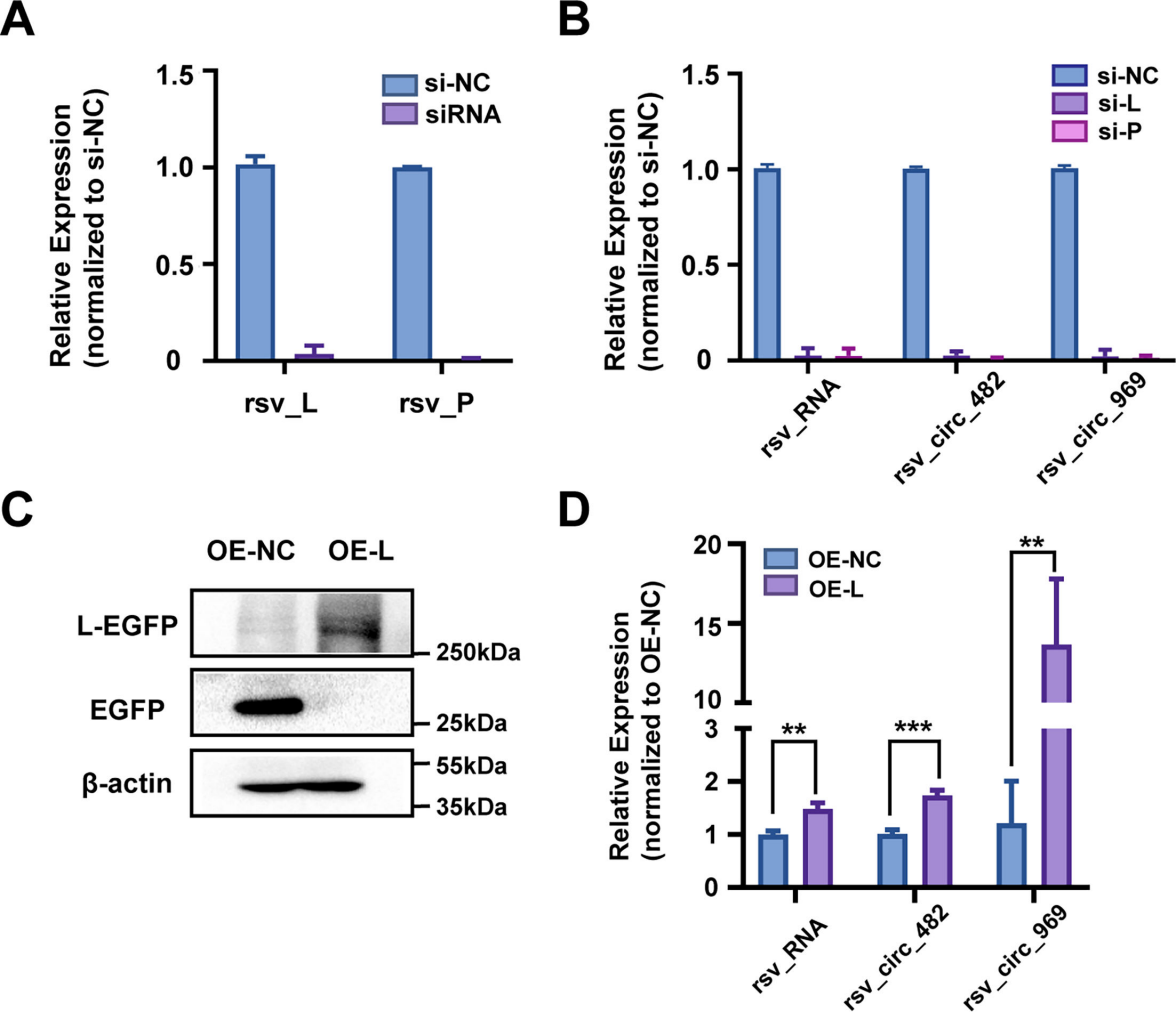
Viral L protein is involved in viral circRNA production. (**A and B**) Effect of L and P knockdown on RSV circRNA production. HEp-2 cells were transfected with pooled siRNAs targeting L or P and then infected with RSV (MOI = 1). In panels **A** and **B**, the efficiency of siRNAs and the effect of siRNAs on rsv_RNA and rsv_circ_482/969 expression levels were assessed by qRT-PCR, respectively. (**C and D**) Effect of L overexpression on RSV circRNA production. HEp-2 cells were transfected with L-EGFP overexpression plasmid and then inoculated with RSV (MOI = 1). In panel **C**, the protein levels of L-EGFP were analyzed by WB. In panel **D**, the effect of L-EGFP overexpression on rsv_RNA and rsv_circ_482/969 expression levels was determined through qRT-PCR. OE, overexpression. **, *P* < 0.01; ***, *P* < 0.001. For all panels, NC means negative control.

The effect of L polymerase on RSV circRNA production was first explored using RNA interference. L functions by interacting with its cofactor, P protein. Therefore, we designed small interfering RNAs (siRNAs) targeting both L and P. HEp-2 cells were transfected with pooled siRNAs prior to RSV infection and collected at 48 hpi. As expected, the pooled siRNAs targeting L or P effectively reduced the levels of their respective mRNAs (Fig. 5A). Compared to si-NC, RSV RNA levels were significantly decreased by both L and P silencing. Under these conditions, both rsv_circ_482 and rsv_circ_969 showed similar reduction upon si-L and si-P knockdown (Fig. 5B). We further investigated whether L overexpression affects RSV circRNA production. HEp-2 cells were transfected with L-EGFP-overexpressing plasmid, followed by inoculation with RSV viruses. The overexpression of L protein was verified by EGFP fluorescence (data not shown) and WB (Fig. 5C). Further results showed that L-EGFP overexpression increased RSV RNA levels (Fig. 5D) and similarly affected rsv_circ_482 and rsv_circ_969 RNA levels (Fig. 5D). These results indicate that interfering with the expression of RdRp L affects viral circRNA production, similar to its effect on RSV RNA synthesis.

Collectively, these results indicate that RSV L protein is involved in viral circRNA production.

### Cellular HSP70/90 proteins are involved in viral circRNA production as co-factors

**Fig 6:**
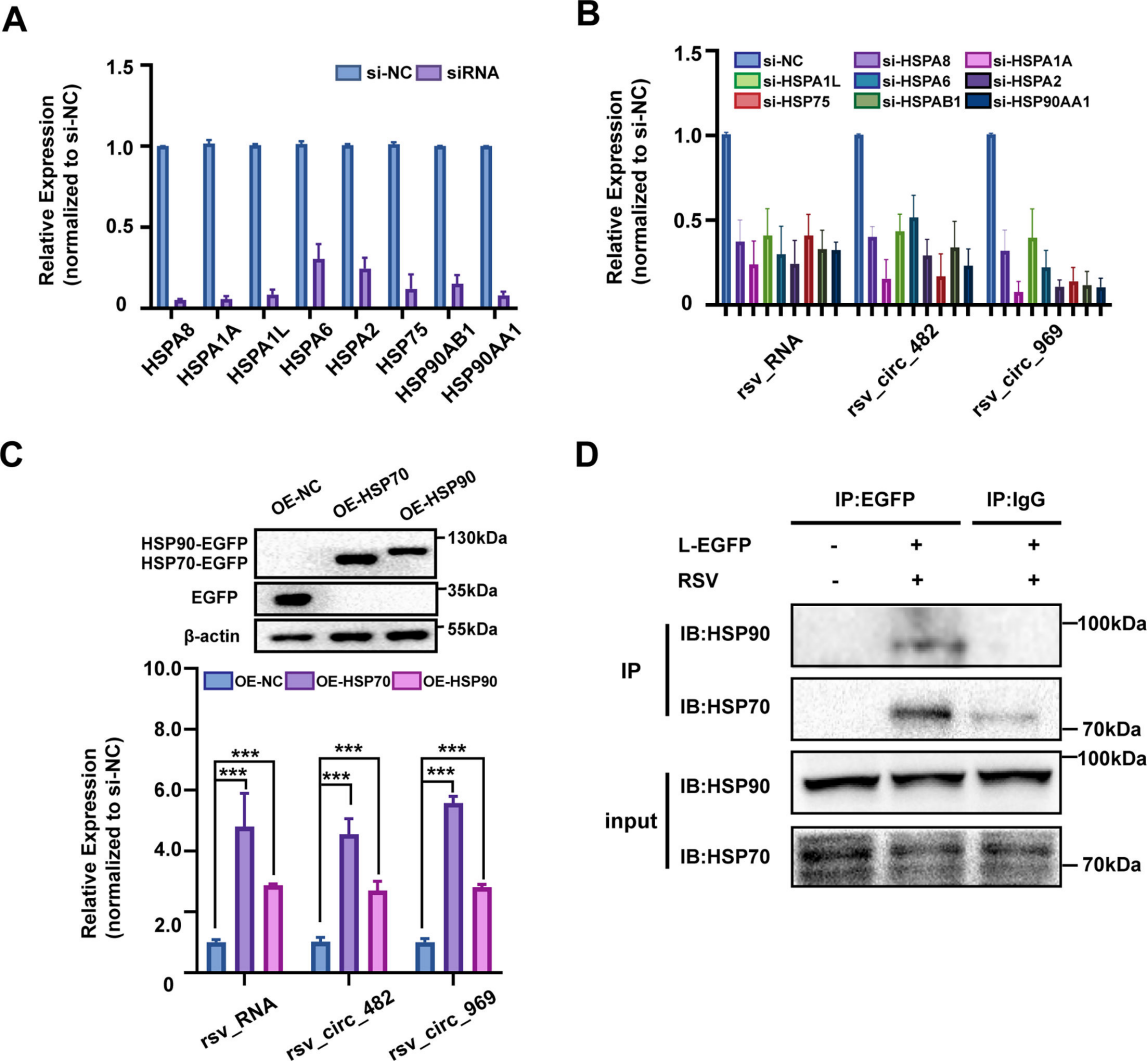
Cellular HSP70/90 proteins are involved in viral circRNA production. (**A and B**) HEp-2 cells were transfected with three pooled siRNA for each HSP70/90 isoform and then subjected to RSV infection (MOI = 1). The efficiency of siRNAs and the effect of siRNAs on rsv_RNA and rsv_circ_482/969 expression levels were analyzed through qRT-PCR. (**C**) HEp-2 cells were transfected with HSP70-EGFP or HSP90-EGFP plasmid and then infected with RSV (MOI = 1). The protein levels of HSP70/90 were analyzed by WB, and the effect of HSP70/90 overexpression on rsv_RNA and rsv_circ_482/969 expression levels was analyzed through qRT-PCR. OE, overexpression. ***, *P* < 0.001. (**D**) CO-IP assay was conducted to determine the interaction of HSP70/90 with RSV L protein in BHK21 cells transfected with pL-EGFP plasmids. Cell lysates were subjected to IP with an anti-EGFP antibody and then immunoblotted with anti-HSP70/90 antibody. For all panels, NC means negative control.

The effect of cellular HSP70/90 proteins on RSV circRNA levels was further examined. HEp-2 cells were transfected with siRNAs targeting HSPs or plasmids overexpressing HSPs, followed by RSV inoculation. Cells were then collected at 48 hpi for analysis. Some isoforms such as HSPA1L are expressed at low levels under normal conditions; the siRNA knockdown experiments were conducted in RSV-infected cells, where the HSP isoforms are known to be robustly upregulated, making them functionally relevant targets. Five HSP70 isoforms and three HSP90 isoforms were identified in the MS experiment (Table 1). In the siRNA interference experiments, we designed corresponding siRNAs for all eight HSPs, and siRNA specificity was confirmed by demonstrating a lack of off-target effects on other isoforms. Results showed that these specific siRNAs efficiently and respectively decreased the RNA levels of HSP70/90s (Fig. 6A). Knockdown of all eight HSP70/90 proteins also reduced the RNA levels of both rsv_circ_482 and rsv_circ_969 (Fig. 6B). In addition, the overexpression of HSP70 and HSP90 proteins was verified by WB, and overexpression of both HSP70 and HSP90 increased the RNA levels of rsv_circ_482 and rsv_circ_969 (Fig. 6C). As expected, interference with the expression of HSP70/90s also affected RSV RNA levels in these experiments (Fig. 6B and C). These results indicate that HSP70/90s affects viral circRNA accumulation.

The observed effect of HSPs on circRNA abundance could, in principle, result from alterations in either synthesis or stability. To directly disentangle these two mechanisms, we performed experiments using a reconstitution circRNA synthesis system, in which viral circRNA is produced exogenously and N, P, and L proteins are co-overexpressed to mimic RSV-induced IBs. Our results with this system showed that overexpression of both HSP70 and HSP90 increased RSV circRNA levels (Fig. S1A), and HSP70 knockdown significantly decreased RSV circRNA levels (Fig. S1B). Since RSV replication is not involved in the system, the observed effect can be attributed to the specific regulatory role of HSPs on viral circRNA synthesis. Additionally, the combination of the RSV L polymerase inhibitor JNJ-8003 (30) with HSP70 overexpression demonstrates that HSP70 can restore viral circRNA levels, even when canonical RSV replication is suppressed (Fig. S1D). These results further suggest that HSPs can directly regulate circRNA abundance while simultaneously influencing RSV replication, which in turn indirectly modulates circRNA levels. Both HSP90 and HSP70 have been reported to be involved in RNA replication of nsNSV by interacting with L polymerase (7). We further investigated whether HSP70/90s directly interact with RSV L proteins using co-IP experiments. BHK21 cells were transfected with L-EGFP and then infected with RSV. Cell lysates were then subjected to IP using anti-EGFP or anti-IgG antibodies and immunoblotted using anti-HSP70 or anti-HSP90 antibodies. Results showed that L-EGFP co-immunoprecipitated with both HSP70 and HSP90 (Fig. 6D), revealing that L interacts with HSP70 and HSP90 in the context of RSV infection.

Collectively, these results indicate that cellular HSP70/90 proteins are involved in viral circRNA production as co-factors through interaction with L proteins.

### The flanking AT/TA sequence of RSV circRNAs functions as a *cis*-acting element

It is known that the canonical GT/AG donor/acceptor sequence flanking eukaryotic circRNAs plays a role in both circRNA generation and mRNA transcription as a *cis*-acting element (28). Our previous study showed that RSV circRNAs predominantly contain an AT/TA flanking sequence, based on the probability distribution of flanking signals in 1,254 viral circRNAs, with the CG/GC sequence being the least frequent (23). The present study shows that RSV circRNA synthesis is similar to RSV RNA synthesis: both processes occur in IBs and require RdRp-L and HSPs. These findings prompted us to investigate whether the AT/TA flanking sequence plays a role in circRNA production and, by extension, in RSV RNA synthesis.

**Fig 7:**
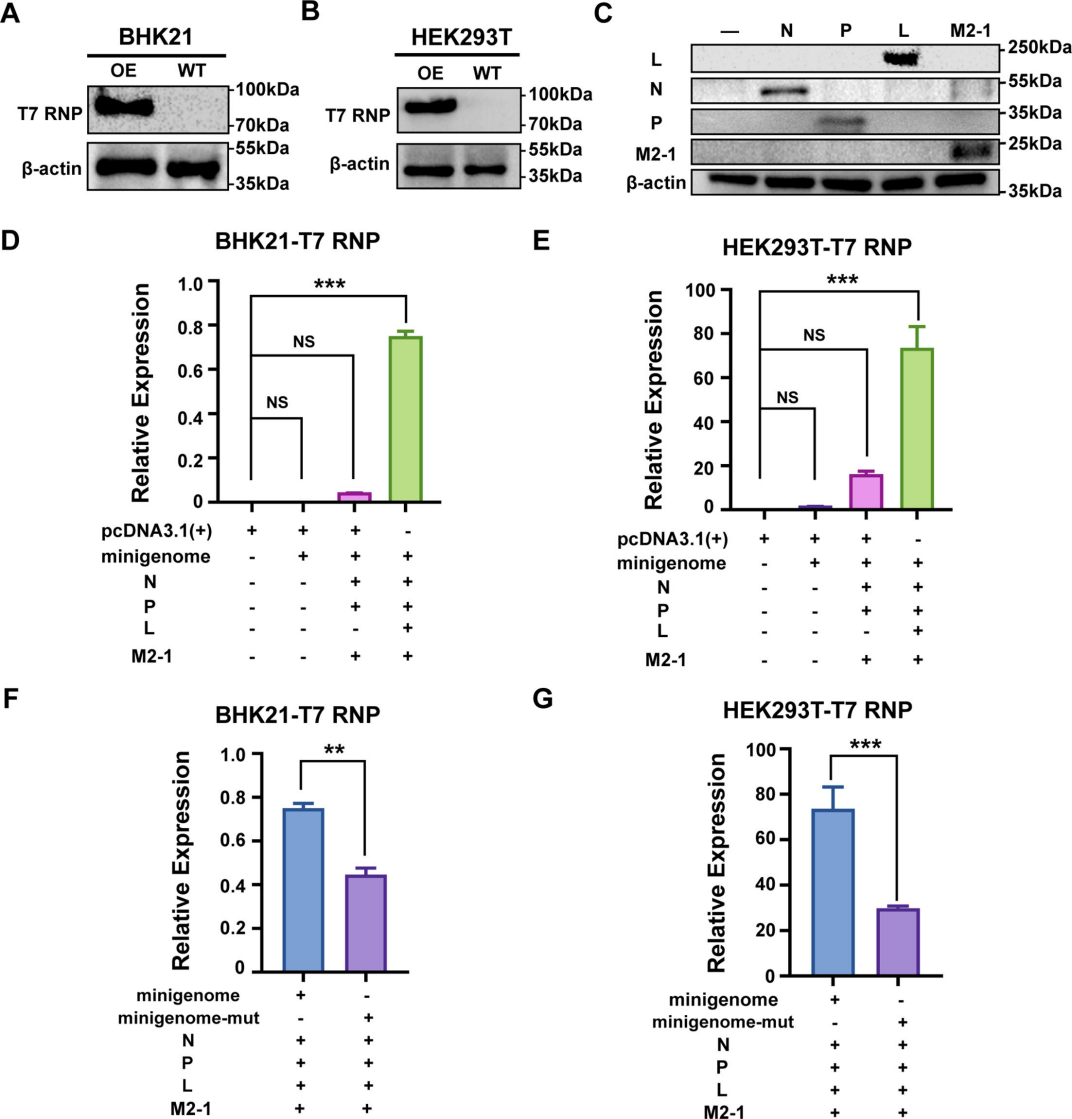
The flanking AT/TA sequence of RSV circRNAs functions as a *cis*-acting element. (**A and B**) Validation of T7 RNP protein expression in BHK21- and HEK293T-derived stable cell lines by WB. (**C**) The expression of support plasmids, pN, pP, pL, and pM2-1, which are required for reverse genetics system, was analyzed by WB. (**D and E**) RSV RNA levels were determined by qRT-PCR at 48 h after co-transfecting BHK21-T7 TNP cells or HEK293T-T7 RNP cells with minigenome replicon and support plasmids. (**F, G**) Effect of flanking sequence mutation on the RSV RNA levels was determined by qRT-PCR in the reverse genetics system described in panels **D and E**. For panels **B** to **E**, *P* values are as follows: **, *P* < 0.01; ***, *P* < 0.001; NS, no significance.

To investigate whether the AT/TA flanking sequence is involved in viral RNA synthesis, we established a T7 promoter-based reverse genetics system for the rescue of RSV, in which the RSV minigenome replicon and four support plasmids were coexpressed. BHK21- and HEK293T-derived cell lines stably expressing T7 RNA polymerase (RNP) were generated and verified by WB (Fig. 7A and B). Four support plasmids expressing the N, P, L, and M2-1 proteins were also verified by WB (Fig. 7C). Then, results showed that RSV RNA synthesis based on the minigenome replicon was successfully carried out and measured by qRT-PCR in the presence of all four functional support proteins. In contrast, RSV RNA synthesis was significantly reduced in the absence of L proteins in both BHK21- and HEK293T-derived T7 RNP stable cell lines (Fig. 7D and E), confirming that RdRp-L protein was required to ensure viral RNA synthesis. We further mutated the flanking sequence of the minigenome replicon to the CG/GC sequence. The results showed that RSV RNA synthesis was significantly impaired by this mutation in both cell lines (Fig. 7F and G), highlighting the critical role of the AA/TA sequence in viral RNA synthesis.

In conclusion, these results suggest the flanking AT/TA sequence of RSV circRNAs acts as a *cis*-acting element in viral RNA synthesis.

## DISCUSSION

This study is the first to investigate the production mechanism for viral circRNAs encoded by RSV. First, from the perspective of protein components, we found that both the viral L polymerase and cellular HSP70/90 proteins associate with viral circRNAs, based on proteomic analysis of rsv_circ_482/969 pulled-down lysates. We further validate that rsv_circ_482/969 associates with L polymerase and HSP70/HSP90 proteins by RIP. These suggest that the viral L and cellular HSP70/90 proteins interact with viral circRNAs to play a certain function, probably acting as interactors to participate in the biogenesis of rsv_circ_482/969. Then, from the perspective of the synthesis site, we found that the biogenesis of RSV circRNA occurs in IBs, and the colocalization of rsv_circ_482/969 with L and HSP70/HSP90 within IBs confirmed their role in biogenesis of viral circRNA. Moreover, consistent with the above results, interfering with the expression of L and HSP70/HSP90 affects viral circRNA production, and the participation of HSP70/HSP90 in circRNA production probably functions as co-factors that interact with L proteins. Finally, we demonstrated that the flanking AT/TA sequence of RSV circRNAs functions as a *cis*-acting element in RNA synthesis.

Our findings indicate that L polymerase participates in the biogenesis of RSV circRNAs as a *trans*-acting viral factor. Compared to host cellular proteins, L polymerase likely plays a key catalytic or enzymatic role. It is well established that RSV RNA synthesis depends on RdRp, which consists of the L protein and its co-factor, P protein. In detail, the L protein contains all enzymatic activities required for RNA synthesis, while the P protein coordinates the activity of L and interacts with multiple proteins to facilitate RNA synthesis (31). The present study further expands our contention that, in addition to RSV RNA synthesis, the synthesis of RSV circRNAs also requires RdRp. As the catalytic core of RdRp, RSV L functions as a multifunctional enzyme containing three distinct enzymatic domains, namely, the RdRp domain, the cap (Cap) addition domain, and the cap methylation domain. Besides the three functional domains, the RSV L also contains two structural domains: the connector domain and the C-terminal domain (32). Among the three enzyme activity domains of L protein, RdRp activity domain is hypothesized to play a fundamental role in circRNA production since ribonucleotide polymerization is the cornerstone of circRNA generation, which is similar to RSV RNA synthesis. However, RSV circRNA synthesis is thought to require the involvement of additional ligases that link 3′ and 5′ ends, which differs from conventional RSV RNA synthesis. It has been reported that RNA cap enzymes, together with RNA ligases and DNA ligases, make up the nucleotide transferase superfamily (33). Given that the Cap domain of L protein and RNA ligase belongs to the same protein superfamily, whether the Cap domain of L protein possesses RNA ligase activity or if other domains contribute to RNA ligase activity, thus mediating the circularization of viral circRNA, remains to be further explored. Additionally, recent research by Yang and colleagues demonstrated that the viral exoribonuclease nsp14 of murine hepatitis virus (MHV) is required for the biogenesis of viral circRNAs (34), further emphasizing the crucial role of viral proteins in the synthesis of viral circRNAs.

RNA viruses typically utilize a limited set of viral proteins to interact and coordinate with host proteins, thereby facilitating viral infection and replication. HSPs belong to the largest family of chaperones. HSP90 is an abundant chaperone in all eukaryotic cells (35). Among different isoforms, HSP90α (HSP90AA1) and HSP90β (HSP90AB1), identified in the current study, are the most prevalent in humans (7). HSP70 accounts for the majority of molecular chaperones in cells (35); the HSP70 isoforms identified in the present study, such as HSPA1A, HSPA2, HSPA6, and HSPA8, are commonly located in the cytosol (7). Given their high abundance and cytoplasmic localization, it is not surprising that RSV, an RNA virus that replicates in the cytoplasm, makes use of these HSP70/90 isoforms for circRNA production. Our experimental results, along with previous studies, observe that both HSP70 and HSP90 interact with the viral L protein in the context of viral replication and circRNA synthesis. Their commonality lies in the fact that they function by interacting with and regulating the activity of L protein. It remains unclear whether they work via distinct mechanisms or overlapping functions; however, it is known that they can act cooperatively and form a network to assist in the correct folding of substrate protein to achieve functional conformation. From the perspective of functional domains, HSP70 comprises two domains: the N-terminal nucleotide-binding domain (NBD) and the C-terminal substrate-binding domain (SBD). In contrast, HSP90 consists of three domains: the N-terminal domain (NTD), the middle domain (M domain), and the carboxy-terminal domain (CTD). HSP70 primarily assists in the folding of newly synthesized proteins through its C-terminal SBD, whereas HSP90 typically receives client proteins from HSP70 chaperone system and accomplishes mature protein stabilization via its M domain (36). Previous studies have reported that HSP70 and HSP90 are involved in RNA replication in various RNA viruses (7, 37). In the context of RSV replication, Munday et al. reported that HSP90 is critical for L protein function and stability, and HSP70 helps the polymerase remodel the nucleocapsid to enable efficient RNA synthesis (11). In summary, while both HSP70 and HSP90 interact with the viral L protein to support viral replication and circRNA synthesis, they likely employ distinct molecular mechanisms to form the integrated HSP70/90 chaperone network, as evidenced by functional domains and studies. Additionally, our results showed that HSPs can directly regulate circRNA biogenesis beyond any indirect effect from global polymerase change while simultaneously influencing RSV replication, which in turn indirectly modulates circRNA levels. Therefore, their contributions in these processes are both independent and interconnected. In conclusion, our findings, along with previous studies (8, 10, 11), indicate that both RSV RNA synthesis and circRNA production require the assistance of HSPs as molecular chaperones. It is noted that the observed effect of HSPs on circRNA abundance could stem from altered synthesis or stability. While our consistent lines of evidence provide direct evidence for a synthesis-level effect, we acknowledge that, within the complex environment of a full viral infection, HSP70/90 may also contribute to circRNA stability, which is worthy of further study in the future.

In terms of cellular localization, most circRNAs are exported from the nucleus after biogenesis and eventually distributed in the cytoplasm, with the exception of intron-containing circRNAs, which are localized in the nucleus (38). In this study, we demonstrate for the first time that RSV circRNAs localized in RSV-induced cytoplasmic IBs. Rincheval et al. identified that IBs are sites of viral RNA synthesis through nascent viral RNA staining with 5EU dye in their study (9). Since 5EU did not distinguish between mRNA, circRNA, and genomic RNA, we extend the concept of viral RNA synthesis to include circRNA synthesis and hypothesize that IBs serve as sites for RSV circRNA synthesis. In the current study, we confirm that the IBs are indeed sites of RSV circRNA synthesis through circRNA staining. Our further research found that these IBs harbor viral and cellular proteins involved in the generation of viral circRNA, namely RSV RdRp and host HSPs. It is now known that nsNSV concentrates RNA synthesis within specialized IBs, also referred to as biomolecular condensates (BCs), which maintain high local concentration of specific macromolecules involved in viral transcription and replication. Similar to the case with RNA synthesis of nsNSV, the specific localization of RSV circRNA in IBs is supposed to demonstrate several promoting effects for circRNA synthesis from the point of view of BCs (39, 40). First, the high local concentration of molecules, including template RNA and catalytic L protein, within condensates can activate biochemical reactions. Second, the chaperone environment of HSPs within condensates can stabilize the conformations of RdRp biomolecules, potentially enhancing their activity and stabilizing enzyme interactions with their substrates. Third, selective sequestration of certain molecules, such as pattern recognition receptors and antiviral effector molecules, enables condensates to escape or suppress host defense mechanisms.

With regard to *cis*-acting elements, most circRNAs from eukaryotic cells are generated through back-splicing, which broadly belongs to alternative splicing and depends on the interactions between the spliceosome machinery and the well-characterized GT/AG *cis*-elements. The spliceosome is a dynamic and sophisticated complex composed of several small nuclear ribonucleoproteins (snRNPs) along with a large number of additional proteins. Rach snRNP contains one snRNA and several proteins. The splicing process initiates with the direct binding of the 5′ splice site (GT) to U1 snRNP and ends with the cleavage of the 3′ splice site (AG) followed by RNA ligation (41). Cao’s study on the splicing of viral circRNAs demonstrated that these circRNAs utilize a significantly higher proportion of non-canonical back-splicing signals (42). Our previous study demonstrated that RSV circRNAs have flanking AT/TA donor/acceptor sequences, rather than canonical GT/AG splicing sequences (23). In this study, we mutated the flanking AT/TA sequence into CG/GC sequence, which had the lowest probability distribution, and found that this mutation significantly affects RSV RNA synthesis, suggesting that the AT/TA sequence functions as a *cis*-acting element. It is known that each RSV gene begins with a 9-nucleotide (nt) gene-start (GS) signal and terminates with a 12–14-nt gene-end (GE) signal. During transcription, the L polymerase copies the genes into their corresponding mRNAs through a sequential start–stop process guided by the GS and GE signals (5). Interestingly, L polymerase of RNA viruses exhibits homologous similarity to the pre-mRNA splicing factor 8 in the eukaryotic spliceosome (43), and this similarity has long been of significant research interest. For RSV, whether the AT/TA flanking sequences participate in viral circRNA or RNA synthesis by guiding or interacting with L polymerase, or whether they function via RNA-protein macromolecular complexes similar to eukaryotic spliceosomes, containing L polymerase and other viral/host factors, remains an intriguing topic that deserves further investigation.

**Fig 8:**
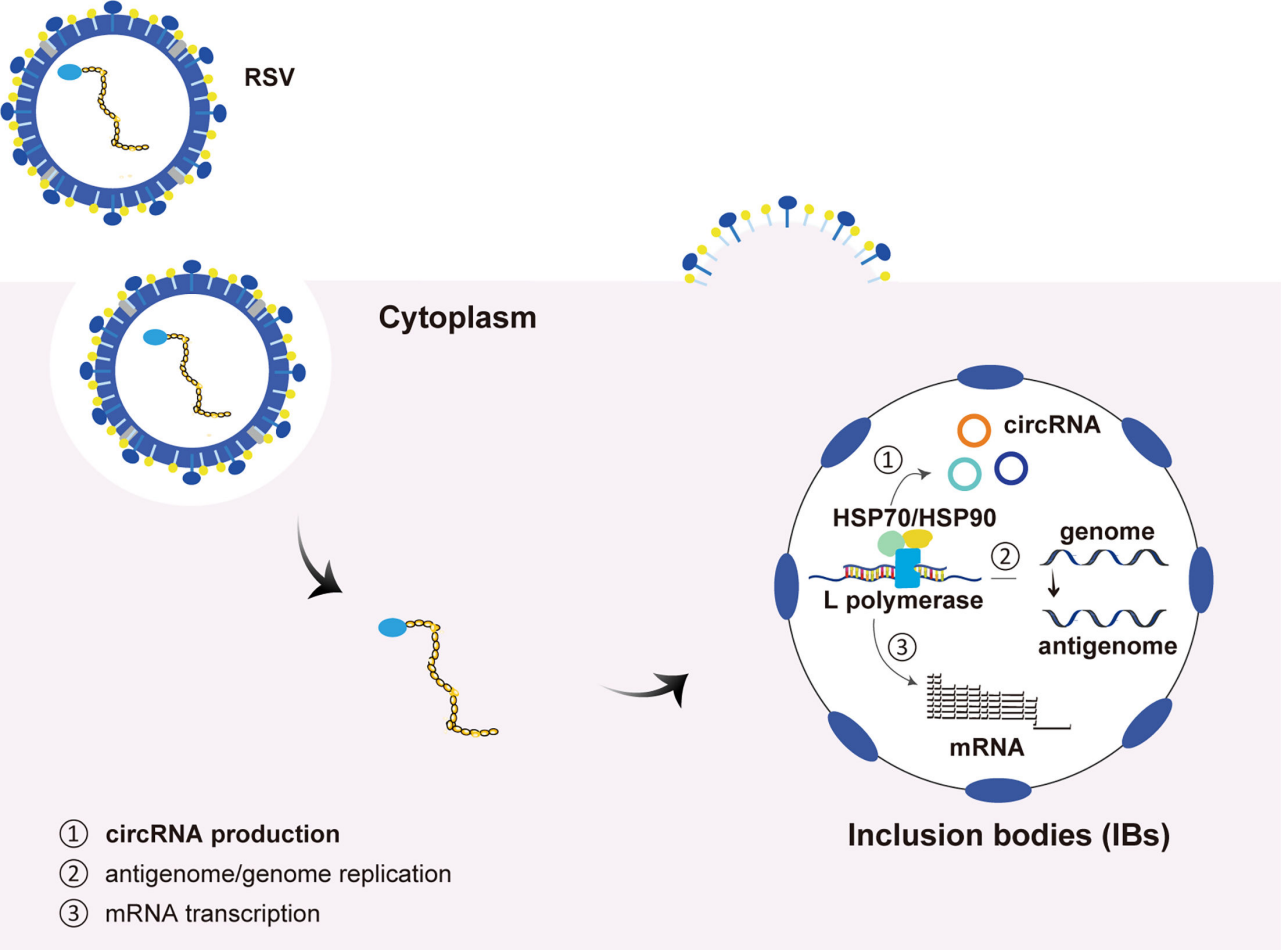
A proposed model of the production mechanism of RSV circRNAs. The pink background indicates cytoplasm of host cells. Similar to the canonical RSV RNA synthesis (including antigenome/genome replication and mRNA transcription), the biogenesis of RSV circRNAs occurs in RSV-induced IBs. Viral L polymerase (blue) and host HSP70/90 (cyan/yellow) proteins are recruited to IBs, synergistically participating in the circRNA synthesis as *trans*-acting factors: L polymerase binds to the RSV genome and plays important catalytic or enzymatic roles, whereas HSP70/90s function as assistant molecular chaperones.

Overall, we propose a model that illustrates the production mechanism of RSV circRNAs (Fig. 8). In this model, similar to the canonical RSV RNA synthesis (including antigenome/genome replication and mRNA transcription), the biogenesis of RSV circRNAs occurs in IBs; viral L polymerase and host HSP70/90s synergistically participate in the circRNA synthesis as *trans*-acting factors; L polymerase binds to the RSV genome and plays important catalytic or enzymatic roles, whereas HSP70/90s function as assistant co-factors that create a permissive environment for circRNA biogenesis within the IBs. In addition, the flanking AT/TA sequence of RSV circRNAs functions as a *cis*-acting element. Taken together, our study identifies *trans*-acting factors and *cis*-acting elements required for the biogenesis of RSV circRNAs and reveals novel layers of host-RSV interactions. A comprehensive understanding of the crosstalk among RSV, circRNA, L polymerase, HSP70/90 proteins, and IBs is crucial for identifying novel antiviral targets. Several antiviral strategies against RSV are in preclinical or clinical development (44). These include small molecules targeting viral proteins, such as L polymerase. Targeting critical host proteins is also a promising strategy. In addition, recent studies have shown that cyclopamine and its analog inhibit RSV replication by disrupting and hardening IB condensates (45). The demonstrated involvement of L polymerase and host HSP70/90 proteins in RSV circRNA biogenesis at IBs further supports the idea that these protein factors and condensates are promising anti-RSV therapeutic targets.

## MATERIALS AND METHODS

### Cells and virus

HEK293T cells (ATCC), HEp-2 cells (ATCC, CCL-23), and BHK21 cells (ATCC) were cultured in Dulbecco’s modified Eagle’s medium (DMEM; Gibco, C11965500BT) supplemented with 10% (vol/vol) fetal bovine serum (FBS) and 1% (vol/vol) penicillin-streptomycin and maintained at 37°C in a humidified 5% CO2 atmosphere. BHK21- and HEK293T-derived cell lines that constitutively express T7 RNP were constructed by infecting cells with a lentivirus carrying the coding sequence of T7 RNA polymerase. These cells were maintained in the medium described above and supplemented with 0.3 μg/mL of puromycin. RSV (A2 strain) was grown in HEp-2 cells for large-scale preparation. For viral infections, cells were washed with phosphate-buffered saline (PBS) and incubated with the viral inoculum at the indicated multiplicity of infection (MOI) in viral growth medium (DMEM with 2% FBS and 1% penicillin-streptomycin) for 2 h at 37°C. During viral inoculation, the culture was gently rocked every 30 min to ensure even distribution of the inoculum. Finally, the appropriate amount of fresh medium was added. Virus titers were measured by counting plaques formed by serial dilutions of the virus supernatants added on HEp-2 cell monolayers as previously described (23).

### Plasmid construction

Expression plasmids of RSV L, P, N, and M2-1 proteins were designated as pL, pP, pN, and pM2-1. Plasmid p-minigenome carries a dicistronic minigenome derived from RSV genome (RSV strain A2, GenBank accession number M74568.1), consisting of partial NS1 (nucleotides 45 to 98) and partial L (nucleotides 8489 to 15067), coupled with leader region (nucleotides 1 to 44) and trailer region (nucleotides 15068 to 15222). Based on the p-minigenome, we generated a mutant plasmid through site-directed mutagenesis that has four nucleotides substituted at positions 43 to 44 (AT to CG mutation) and 15069 to 15070 (TT to GC mutation), which is called p-minigenome-mut. Plasmids expressing RSV_N, RSV_P, and RSV_L that were tagged with EGFP, designated as N-EGFP, P-EGFP, and L-EGFP, were constructed by inserting EGFP coding sequence into the vector pcDNA3.1(+). pHSP70-EGFP and pHSP90-EGFP were constructed by inserting coding sequence of either HSP70 (NM_001271971.2) or HSP90 (NM_001271971.2) into the vector p-EGFP-C1. The rsv_circ_482/969, rsv_circ_482/969-MS2, and MCP overexpression vectors were constructed by Geneseed (Guangzhou, China).

### Antibodies

The following primary antibodies were used throughout our study: anti-Flag (Kingsray, A00170), rabbit anti-HSP70 (Proteintech, 10654-1-AP), rabbit anti-HSP90 (Cell Signaling Technology, 5087), polyclonal rabbit anti-EGFP (Abcam, ab290), anti-GFP (Santa Cruz, sc-9996), rabbit anti-EGFP (Sino Biological, 16118), goat anti-RSV (Merck Millipore, AB1128), anti-GAPDH (Beyotime, AF0006), and anti-β-actin (Bioworld, AP0060). The secondary antibodies we used are as follows: goat anti-mouse (Beyotime, A0216), goat anti-rabbit (Beyotime, A0208), and mouse anti-goat (Santa Cruz, sc-2354).

### circRNA pull-down assays

HEK293T cells were co-transfected with rsv_circ_482/969-MS2 and MCP overexpression plasmids using Lipo8000 (Beyotime) according to the manufacturer’s instructions. Cells co-transfected with rsv_circ_482/969 and MCP-NC plasmids were used as negative controls. Cells were then infected with RSV 24 h post-transfection and harvested at 48 hpi. Then, the cell lysates were incubated with Flag-binding magnetic beads for 2 h at 4°C. The magnetic beads were washed 10–15 times with wash buffer. The proteins and circRNAs bound to the beads were then collected with elution buffer and further analyzed by mass spectrometry (Geneseed, Guangzhou, China), WB, and qRT-PCR assay. Specifically, the protein score in proteomics analysis is generated by Mascot software by matching mass spectrometry data (the experimental mass-to-charge ratio patterns) against protein databases and evaluating the quality of these matches.

### RNA-binding protein immunoprecipitation assay (RIP)

RIP kit (Milipore, No.17-701) was applied following the manufacturer’s instructions. In brief, BHK21 cells were individually transfected with HSP70-EGFP, HSP90-EGFP, or L-EGFP plasmids and subjected to RSV infection (MOI = 1) 24 h post-transfection. At 48 hpi, cells were harvested and lysed with RIP lysis buffer. The lysates were incubated with magnetic beads coated with either anti-EGFP antibodies or control anti-IgG antibodies at 4°C overnight. The beads were then washed and treated with proteinase K. Finally, RNA was purified through phenol‒chloroform‒isoamyl alcohol method and analyzed using qPCR.

### Fluorescent *in situ* hybridization (FISH) and confocal microscopy

HEK293T cells cultured on glass coverslips in 24-well plates were transfected with pN-EGFP, pP-EGFP, pL-EGFP, pHSP70-EGFP, or pHSP90-EGFP plasmids and subjected to RSV infection (MOI = 0.5) 24 h post-transfection. At 48 hpi, the cells were fixed with 4% paraformaldehyde (vol/vol) for 10 min at room temperature (RT) and permeabilized with cold PBS containing 0.5% Triton X-100 (vol/vol) for 5 min at 4°C. Then, cells were washed three times with PBS for 5 min each, preparing them for subsequent probe detection. According to the manufacturer’s recommendation for the Ribo Fluorescent *In Situ* Hybridization Kit (RN: R11060.7), cells were incubated with pre-hybridization buffer (containing 1% blocking solution) for 30 min at 37°C. The pre-hybridization buffer was then discarded, and cells were incubated with a hybridization mix containing specific probes in the dark at 37°C overnight. Then, cells were rinsed three times with hybridization wash buffer I (4× SSC), followed by a single rinse with hybridization wash buffer II (2× SSC) and buffer III (1× SSC). All washing procedures were conducted at 42°C in the dark. Nuclei were then stained with 1× DAPI for 10 min in the dark and washed with PBS. Finally, the coverslips were fixed on slides, and images were captured on a confocal microscope (Zeiss, LSM 800).

### Western blotting

Proteins were extracted with RIPA buffer (Thermo Fisher, 89900), and the concentration of total proteins was determined using a BCA protein assay kit (Pierce). Each sample (20 μg of protein) was separated by sodium dodecyl sulfate-polyacrylamide gel electrophoresis (SDS-PAGE) and transferred to polyvinylidene difluoride (PVDF) membranes (Merck Millipore) using a Bio-Rad wet transfer apparatus. After blocking with 0.5% non-fat milk in PBST for 1 h at RT, the membranes were incubated at 4°C overnight with a diluted specific primary antibody. Then, the membranes were washed using PBST and probed with either goat anti-mouse or anti-rabbit IgG horseradish peroxidase (HRP)-conjugated secondary antibody for 1 to 2 h at RT on shaker. Signals were developed using chemiluminescent HRP substrate (Merck Millipore) and recorded using an ECL protein detection system (ChemiDoc XRS+).

### Co-immunoprecipitation (co-IP) assays

BHK21 cells were transfected with 16 µg of pL-EGFP and infected with RSV (MOI = 3) 24 h post-transfection. Cells were then washed with cold PBS and lysed with lysis buffer. Following the manufacturer’s instructions, anti-GFP magnetic beads (P2132, Beyotime) and mouse IgG magnetic beads (P2171, Beyotime) were washed three times with TBS. The magnetic beads were then added to the protein samples and incubated at 4°C overnight. After incubation, the mixture was separated using a magnetic rack to remove the supernatant. The remaining magnetic beads were then suspended in SDS-PAGE sample loading buffer (1×) and heated at 95°C for 10 min. Finally, the protein samples were stored at −80°C for subsequent WB analysis.

### Minigenome replicon studies

As described previously (9), HEK293T-T7 RNP and BHK21-T7 RNP cells, exponentially growing in 6-well plates, were respectively transfected with a plasmid mixture containing 1 μg of pN, 1 μg of pP, 0.5 μg of pL and 0.25 μg of pM2-1, and 1 μg of p-minigenome to measure the activity of the replicon.

### RNA extraction and quantitative real-time PCR

RNA was extracted using the RNA-Quick Purification Kit (Esunbio, RN001), according to the manufacturer’s instructions. In brief, cells were lysed with lysis buffer and then mixed with ethanol. RNA was collected and purified using a centrifugal column. cDNA was synthesized from 1 μg of total RNA using the HiScript III All-in-one RT SuperMix Perfect for qPCR system (Vazyme), according to the manufacturer’s instructions. For each qPCR mixture, 2 µL of diluted cDNA, 0.8 µL of primer pair, and 10 µL of Pro Universal SYBR qPCR Master Mix were combined to make up a final volume of 20 µL with nuclease-free water. qPCR was performed on ABI 7500 system (Thermo Fisher). The housekeeping GAPDH or 18S rRNA gene served as an internal control.

## SUPPLEMENTAL MATERIAL

Fig. S1 mbio.03980-25-s0001.tif HSPs can regulate circRNA expression independently.

Legends mbio.03980-25-s0002.docx Supplemental table legend and figure legend.

Table S1 mbio.03980-25-s0003.xlsx Identified proteins.

ASM does not own the copyrights to Supplemental Material that may be linked to, or accessed through, an article. The authors have granted ASM a non-exclusive, world-wide license to publish the Supplemental Material files. Please contact the corresponding author directly for reuse.
