# Supplementary figures and images for: Involvement of L polymerase and heat shock proteins in the biogenesis of viral circular RNAs derived from respiratory syncytial virus
Source: mBio. 2026 Feb 12;17(3):e03980-25. doi: 10.1128/mbio.03980-25 (PMC12977508; doi:10.1128/mbio.03980-25)

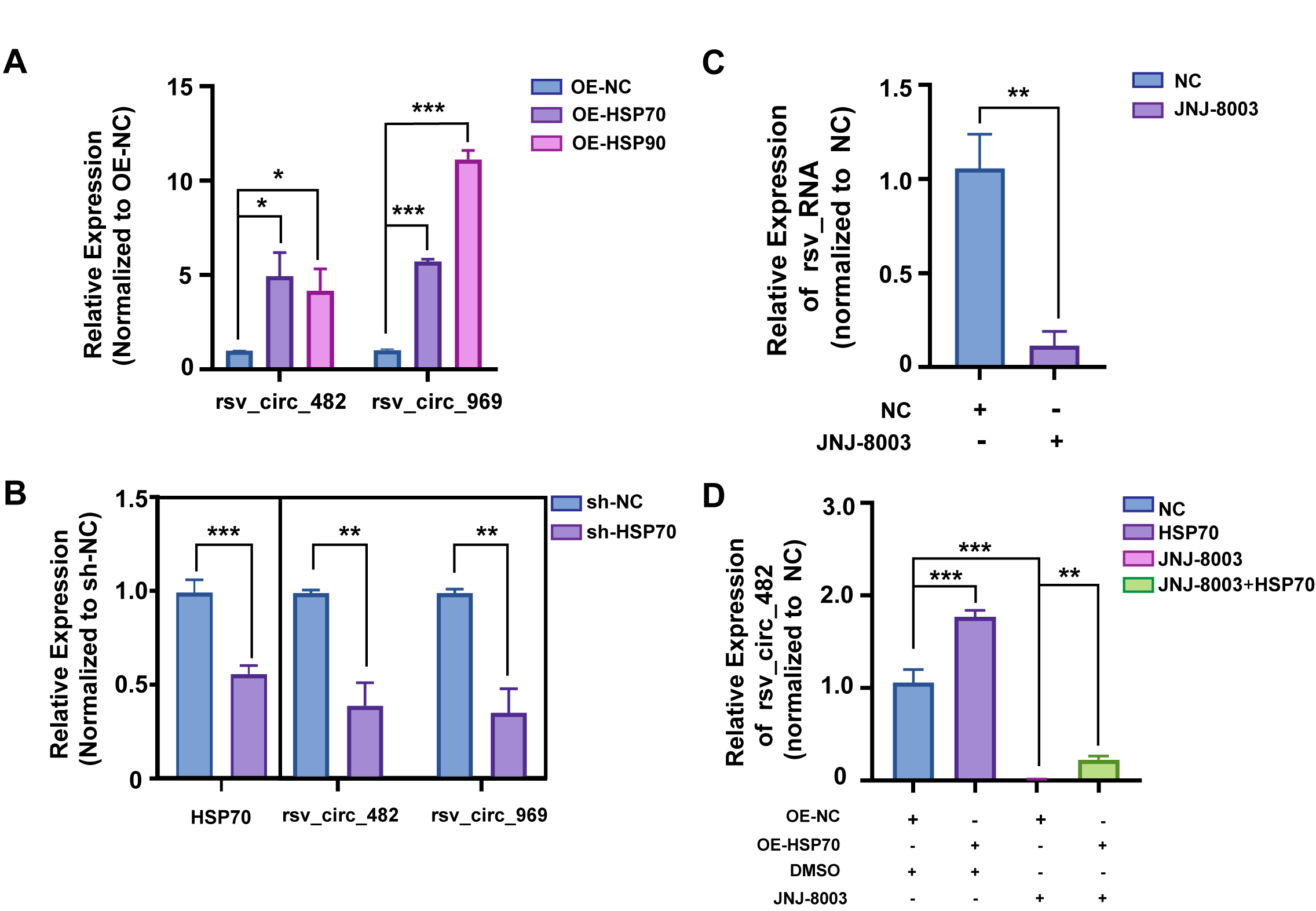

Supplement: Fig. S1 — HSPs can regulate circRNA expression independently. [file mbio.03980-25-s0001.tif]
