# Supplementary material for: Involvement of L polymerase and heat shock proteins in the biogenesis of viral circular RNAs derived from respiratory syncytial virus
Source: mBio. 2026 Feb 12;17(3):e03980-25. doi: 10.1128/mbio.03980-25 (PMC12977508; doi:10.1128/mbio.03980-25)
Supplement: Legends — Supplemental table legend and figure legend. [file mbio.03980-25-s0002.docx]

**Supplementary Table Legend**

**Supplementary Table 1: All proteins identified in the circRNA pulldown assays coupled MS proteomics.** We supply all proteins identified in the circRNA pulldown assays coupled MS proteomics, sorting in descending order of protein score and protein cover percentage.

**Supplementary Figure Legend**

**Supplementary Figure 1: HSPs directly regulate circRNA biogenesis beyond any indirect effect from RSV replication.** **(A)** To determine the effect of HSPs on circRNA synthesis, HEK-293T cells were co-transfected with rsv_circ_482/969 overexpression plasmids (to procduce viral circRNA), N, P, and L overexpression plasmids (to stimulate RSV-induced IBs) and HSP70/HSP90 overexpression plasmids. CircRNA levels were determined by qRT-PCR at 48 h post-transfection. **(B)** HEK-293T cells were transfected with shRNAs targeting HSP70 for 48 h and then the viral supernatant was collected. Subsequently, using the same system as in **(A)**, HEK-293T cells were co-transfected with rsv_circ_482/969, N, P, and L overexpression plasmids and then infected with the collected viral supernatant. The knockdown efficiency of shRNAs and the subsequent effect on rsv_circ_482/969 expression levels were assessed by qRT-PCR. **(C)** RSV-infected HEp-2 cells (MOI=1) were treated with either the RSV replication inhibitor JNJ-8003 (1 nM) or DMSO as a vehicle control. The efficiency of JNJ-8003 were confirmed by quantifying RSV RNA levels via qRT-PCR. **(D)** RSV-infected HEp-2 cells (MOI=1) were transfected with either a pcDNA3.1(+) (NC) plasmid or HSP70 overexpression plasmid for 24 h. And then the cells were treated with either DMSO or JNJ-8003 and incubated for additional 24 h. Levels of rsv_circ_482 were then quantified by qRT-PCR to determine the effect of HSP70 on viral circRNA production under conditions of inhibited RSV replication. OE, overexpression. NC, negative control. *, *P* < 0.05; **, *P* <0.01; ***, *P* < 0.001.
